# Conjugate Vaccines Targeting Tumor-Associated Carbohydrate Antigens

**DOI:** 10.3390/vaccines14040287

**Published:** 2026-03-24

**Authors:** Nadine Rosenglick, Géraud Valentin, Kiran Marineni, Euclydes P. Neto, Peter R. Andreana

**Affiliations:** Department of Chemistry and Biochemistry, School of Green Chemistry and Engineering, University of Toledo, 2801 W. Bancroft St., Toledo, OH 43606, USA; nroseng@rockets.utoledo.edu (N.R.); geraud.valentin@utoledo.edu (G.V.);

**Keywords:** conjugate vaccine, immunotherapy, cancer, tumor-associated carbohydrate antigen, vaccine carrier, antigen, T-cell independence

## Abstract

The surface of cancer cells is covered in abnormal carbohydrate antigens that facilitate tumor growth, immune evasion and metastasis. Overexpressed and often specific to cancer cells, these tumor-associated carbohydrate antigens (TACAs) offer a valuable handle for targeted immunotherapy and were soon targeted by TACA–protein conjugate vaccines. Despite good initial results, sTn-KLH conjugate Theratope^®^ failed in clinical trials fifteen years ago for failure to improve life expectancy. This has been attributed to poor immunogenicity, inhomogeneous expression of TACAs within tumors, and vaccine carrier interference. This review covers the two decades of subsequent effort to overcome these limitations and the now large toolbox available to vaccine researchers to improve the outcome of anticancer vaccines: analogues and conformation-locked mimics of TACAs, monomolecular multivalent vaccines, more biologically relevant presentation of TACAs through clusters and glycopeptides, and a new generation of vaccine carriers to reduce carrier interference, immune reaction, or provide simple modular vaccine delivery platforms.

## 1. Introduction

Cancer causes 16.8% of deaths worldwide, second only to cardiovascular diseases [1]. Already dominating in high-income countries, current trends have cancer overtaking heart failure worldwide by the end of the century [2]. Overall, new cases are expected to rise from under 20 million annually to over 35 million by 2050. While mortality has consistently gone down for decades thanks to tremendous research efforts, progress has been stalling for some hard-to-treat cancers, including lung and pancreatic ones [3,4]. Increased incidence, rising cases and current treatment challenges require a paradigm shift for cancer treatment to improve outcomes for patients.

At the forefront of research lies immunotherapy, a class of treatments focusing on activating immune mechanisms against cancer, which has led to major victories in the 21st century, yielding high-efficacy specific treatments with lower side effects as a complement and possible alternative to traditional treatments. Of note, PD-1 checkpoint inhibitors, preventing cancer cells from triggering programmed cell death, have been the highest grossing drugs in recent years, totaling around 20 billion dollars annually [5]. Similarly, other checkpoint inhibitors, reprogrammed CAR-T cells, cytokine treatments, oncolytic viruses, monoclonal antibodies and vaccines have all met widespread clinical success [6,7,8,9,10,11]. The World Health Organization recognizes oncolytic viruses and vaccines as treatment options with the lowest side effects [12].

As cancer cells are part of self, most cancer pathways are also present on healthy cells, and a targeted therapy could either face tolerance due to this widespread expression, rendering the treatment ineffective, or cause serious side effects due to poor targeting. Hence, finding cancer-specific antigens or, at least, heavily overexpressed antigens remains a key challenge in today’s immunotherapy [13].

Among promising immunotherapeutic targets, tumor-associated carbohydrate antigens (TACAs) have emerged as a promising handle for patient-specific treatments. Due to common mutations in glycosidases and glycosyltransferases, the surface of cancer cells is covered in abnormal, truncated glycans. These TACAs, a set of twenty to thirty common structures, are either overexpressed or specific to cancer, and play key roles at all stages of disease progression from immune evasion to metastasis. As such, they have garnered growing interest as vaccine and monoclonal antibody targets [14].

However, like most carbohydrates, TACAs are incapable of processing by MHCII and are thus T-cell independent, making it hard for the immune system to mount a competent cellular immune response towards them. This is bypassed by conjugating the antigen(s) to a more immunogenic carrier, typically a protein. While this approach is widely successful against bacteria and used in several commercially available vaccines, it has not yet been clinically successful against cancer [15,16]. This review discusses glycoconjugate vaccines targeting TACAs, past clinical challenges and current attempts at improving vaccine design to improve clinical outcomes.

## 2. Conjugate Vaccines

### 2.1. Tumor-Associated Carbohydrate Antigens

The destruction of cancer cells by the immune system naturally selects resilient phenotypes that multiply faster or resist apoptosis better until the growth speed of the tumor and its destruction by the immune system reach an equilibrium. Eventually, the constant selective pressure exerted by the immune system favors tumor cells capable of immune evasion, a process called immunoediting [17]. By nature, this mechanism selects for mutations, epigenetic changes or protein expression modifications that favor tumor development.

**Figure 1:**
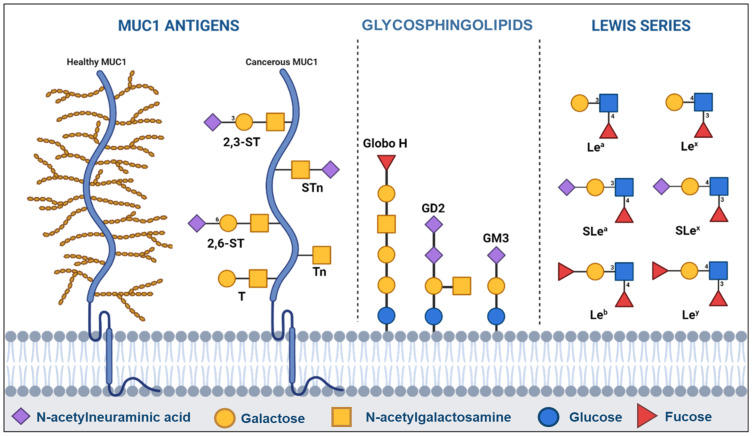
Classification of tumor-associated carbohydrate antigens. Left panel: MUC1 peptides, overexpressed on underglycosylated cancerous mucins, key players of immune evasion. Middle panel: cancer-associated glycosphingolipids, involved in most steps of cancer progression. Right panel: cancer-associated blood group antigens derived from the Lewis blood group, key players of extravasation. Carbohydrate antigens are drawn using the symbol nomenclature for glycans.

Among these modifications, the synthesis of cell surface carbohydrates is one of the most common targets due to the ubiquitous role of carbohydrates in cell communication and immune modulation and to their complex, hence prone to failure, synthesis [18]. This process creates a few oversialylated and fucosylated structures, shared among three major groups (Figure 1).

MUC1 antigens (Tn, sTn, T, sT) are expressed by most adenocarcinomas—mucus-producing tumors—including breast, lung, gastrointestinal or reproductive cancers at the surface of some mucins, a family of large, heavily glycosylated proteins responsible for mucus gel-like consistency [19]. While most mucins are secreted, some, like MUC1, are membrane-bound and protect the cell from its environment, lubricate the cell surface and participate in cell communication [20]. In cancer, MUC1 is severely underglycosylated (Figure 1), exposing the protein backbone and new cancer-specific TACAs. MUC1 and its TACAs have been closely linked to immune evasion through their interaction with immune checkpoints like Siglec receptors, macrophage galactose-binding lectin and DC-SIGN [21,22]. The large difference between healthy and cancerous MUC1, its key role in cancer biology and the presence of new cancer-specific epitopes have ranked MUC1 as the second most important target on the National Cancer Institute (NCI) priority list [23].

Blood group antigens (Le^a^, Le^b^, Le^x^, Le^y^, sLe^a^, sLe^x^), closely related to the ABO antigen system, are more widely expressed, including on MUC1, integrins and growth receptors. The sialylated sLe^a^ and sLe^x^ are common in liver, bladder, colorectal and pancreatic cancer, where they facilitate extravasation and invasion [24], while non-sialylated antigens, especially Le^y^, are linked to increased growth and invasion [25]. Due to their widespread expression, sLe^a^ is used as a diagnostic marker of colorectal and pancreatic cancer [26].

Glycosphingolipids are membrane-bound cell signalers containing the lipid sphingosine. Among them, globosides and gangliosides have been tied to cancer progression. Globosides SSEA-3 and SSEA-4 are normally present during embryonic development, and re-expressed in cancer during regression to a stem cell phenotype [27,28]. Consequently, these oncofetal antigens correlate with poor prognosis. In cancer, they are often fucosylated into cancer-specific Globo H, the most prevalent glycosphingolipid in cancer cells. Globosides are expressed in adenocarcinomas, teratocarcinomas (germ cells) and glioblastomas (brain) and have been linked to angiogenesis, apoptosis resistance and proliferation [29,30,31]. Finally, gangliosides (GM1, GM2, GM3, GD1a, GD2, GD3, GT1b) are a class of sialylated glycosphingolipids used as cell signalers in nerve cells. Due to their prominence in nerve cells, they are usually overexpressed in neuroblastomas (nerve cells), including head, neck, spine and some skin cancers, but can be detected in most tumors [32,33]. Gangliosides have been tied to most steps of cancer progression from growth to immune evasion to metastasis [34]. While some are expressed by healthy cells and only overexpressed in cancer (GM1, GM3), other gangliosides are cancer-specific markers (GD2, GD3). Due to its high expression and cancer-specificity, GD2 is 12th on the NCI prioritization list [23].

The ubiquitous presence of TACAs at the surface of cancer cells, their absence or low presence on healthy cells and their pivotal roles at all steps of cancer progression, from growth to immune evasion to metastasis, designate them as targets of choice for immunotherapy. Consequently, naxitamab and dinutuximab, two monoclonals targeting ganglioside GD2, have recently been approved by the FDA to treat high-risk neuroblastoma [35,36]. More efforts are required to continue developing high-specificity monoclonal antibodies for acute treatment and effective vaccines for long-term remission and relapse prevention against a wide variety of TACAs and cancer types.

However, TACAs are, like all carbohydrates, T-cell independent. While capable of eliciting a weak IgM response by directly engaging the B-cell receptor, carbohydrates cannot generally interact with MHC molecules restricted to peptides. Instead, synthetic carbohydrate-based vaccines are commonly attached to immunogenic carrier proteins as conjugate vaccines. These protein carriers provide the peptide motifs necessary for antigen loading onto MHC-I (via cross-presentation) to elicit CD8^+^ cytotoxic T-cells and MHC-II (via direct presentation) to elicit CD4^+^ T-cells and isotype switching to high-affinity IgGs [37,38].

### 2.2. Traditional Approach to Conjugate Vaccines

Invented in the late 1920s by Avery and Goebel, conjugate vaccines are safe by design as they do not rely on pathogens, are effective at any age, and are excellent at targeting the glycocalyx, normally used by bacteria as a low immunogenicity cover to hide from the immune system. Hence, they have been vastly successful against bacteria, and commercial conjugate vaccines are available worldwide to target *Streptococcus pneumoniae* (Prevnar 20, Capvaxive, Vaxneuvance) [39,40,41], *Haemophilus influenzae* type B (ActHib, Hiberix, PedVaxHIB) [42,43,44], *Neisseria meningitidis* (Menactra, Menveo, Menhycia) [45,46,47], *Salmonella typhi* (Typbar TCV, TYPHIBEV) [48,49] or SARS-CoV-2 (Soberana 02) [50].

The first application of conjugate vaccines to TACAs dates back to Livingston’s group in 1994, who conjugated ganglioside GD3 from clinical isolates to poly-lysine, bovine serum albumin (BSA), keyhole limpet hemocyanin (KLH), malaria T-cell epitope or meningococcal outer membrane complex (OMPC) carriers [51]. Among all tested carriers, KLH exhibited the highest antibody titers. The approach eventually led to the clinical evaluation of GM2-KLH [52]. Concurrently, sTn-KLH construct Theratope^®^ (Biomira, Edmonton, KY, USA) also passed early phase clinical trials [53]. Promising initial results led to further investigations into human serum albumin (GM3-HAS) [54] and a nontoxic mutant of diphtheria toxin, CRM197 (RM2-CRM197) [55], as alternative carriers.

Around 2010, and despite good immunogenicity, both Theratope^®^ and GM2-KLH failed in phase III clinical trials for the lack of increased mean survival. To this day, still, no TACA conjugate vaccine has successfully passed clinical trials, despite the large experience gathered from antibacterial vaccines [15,56]. This unexpected failure has been attributed to the lack of screening for sTn expression in patients, an insufficiently effective carrier, and a targeting limited to only one TACA, not accounting for their heterogeneity on the tumor [15]. The vaccines have also faced an increased risk of immune tolerance to TACAs due to their similarity to self-carbohydrates and their difficulty in bypassing the immunosuppressive tumor microenvironment.

To bypass these problems, a new generation of vaccines is under development, trying to increase immunogenicity and response through different approaches: the use of synthetic TACAs provides purer, better defined and easier to scale antigens than clinical isolates; the presentation of multiple TACAs reduces the risks of immune tolerance and the impact of tumor microheterogeneity; the covalent attachment of toll-like receptor (TLR)- or MHC-binding sequences to the vaccine increases uptake by APCs; and the cluster presentation, conformational blocking and creation of new glycopeptide epitopes tries to better mimic the natural presentation of TACAs.

## 3. Refining Antigen Presentation

### 3.1. Synthetic Antigens

Preparation of the pioneer GM2-KLH vaccines required tedious isolation of the ganglioside. Recent progress in carbohydrate synthesis has opened the way for fully synthetic antigens, yielding reproducible, perfectly homogeneous, pure and easily modifiable antigens. This has paved the way for improved linker chemistry, through customizable conjugation handles, and facilitated structure–activity relationship studies requiring site-specific modification of TACAs (see chemical modification). Danishefsky’s group reported the synthesis of Globo H, fucosyl-GM1, GM2, GD2, GD3 and Le^y^ TACAs, conjugated to KLH [57]. After twenty years of development, Globo H-KLH is currently in phase III clinical trials (NCT03562637) [58].

### 3.2. The MUC1 Peptide

Due to their prominent role in metastasis and immune invasion in adenocarcinomas, MUC1 TACAs Tn, Tf, sTn and sTf have been targeted by numerous vaccine attempts. However, their small size (mono- to trisaccharides) makes them hard to target effectively. As cancerous MUC1 is underglycosylated, exposing the underlying peptide backbone, several vaccines have extended the TACA epitope into a TACA-MUC1 glycopeptide with encouraging results [59,60]. The Kunz group developed a large series of MUC1 peptides bearing Tn and/or sTn antigens with different MUC1 epitopes, antigen densities, and carriers. The final sTn-STAPPA (MUC1 epitope)-tetanus toxoid vaccine yielded the high-affinity, high-specificity anti-sTn monoclonal antibody GGSK I-30 [61,62]. Similarly, Clausen’s group created the 5E5 mAb using a Tn/sTn-MUC1-KLH construct vaccine (Duersbergwerg, Germany) [63]. Finally, Boons’ group demonstrated excellent immune activity by conjugating a Tn-MUC1-derived glycopeptide with a T-helper peptide and a TLR agonist adjuvant in a single self-adjuvanting construct [64].

### 3.3. Chemical Modification

A major benefit of synthetic antigens is the possibility to explore their structure–affinity relationship, refine pharmacokinetics, and lock the conformation to guarantee proper antigen presentation. Fluorine, a bioisostere of the OH group, increases drug half-life and often affinity, and has been placed by Ye’s group at strategic positions of the Tn, T, sTn and GM3 TACAs in KLH conjugates, multiplying antibody titers by up to four compared with natural carbohydrates [65]. Similar results were obtained for a fluorinated KH-1-CRM197 conjugate [66]. Other site-specific modifications, including methylation and chlorination, while better than their natural counterparts, do not outperform fluorinated compounds when tested side by side [67,68]. The glycosidic bond was also replaced by hydrolysis-resistant C-C [69], C-S [70], or C-Se [71] bonds to increase vaccine half-life and, hence, immune response, but a lack of in vivo tests or *O*-glycosylated controls reduces the scope of the work.

Antigen conformation in a vaccine can differ from its conformation on a biologically relevant substrate. As demonstrated by the Martinez-Saez group, the rigid bond between the Tn antigen and its threonine substrate forces it to adopt a ^4^C_1_ conformation [72]. To mimic this, Nativi’s group designed a strained fused polycyclic analogue of the antigen, connecting positions 1 and 2 and mimicking the *O*-threonine linkage to force the antigen to a more biologically relevant conformation [73]. While this approach did not match natural Tn using a five-membered ring, a tricyclic analogue mimicking the peptide bond with a six-membered lactam ring largely outperformed natural Tn in vivo using CRM197 conjugates. A similar sTn derivative has been synthesized and is pending evaluation [74,75]. Similarly, an unnatural iminosugar analogue of Tn linking position 6 to the in-ring nitrogen to form a bicyclic ^4^C_1_-locked analogue elicited four times higher IgG titers than regular Tn in KLH conjugates [76]. Finally, a GM3 analogue, replacing the traditional glycosidic bond between two carbohydrate residues with a lactone to force a biologically relevant pouch conformation proved immunogenic, although without comparison with natural GM3 [77].

### 3.4. Adjuvants

Despite extensive research, there is currently no approved alternative to aluminum salts in conjugate vaccines targeting carbohydrates. Adjuvants routinely used in different vaccine constructs, like QS-21, MPLA, or CpG, only provided marginal improvements to the response of conjugate vaccines in humans [78,79]. Consequently, while safer, more potent adjuvants are still under development, they will not be detailed here, as their transposability to human trials is uncertain at best.

A more promising approach is the direct conjugation of antigens to known adjuvants α-GalCer and MPLA. Using the adjuvant as a carrier guarantees a maximization of the adjuvant effect towards the antigen. MPLA, a non-toxic TLR-4 agonist derived from *Salmonella Minnesota* cell walls, was used as a carrier for TACAs GM3 [80], Globo H [81], sTn and sTn analog sTnNPhAc [82]. All reported vastly increased IgG titers compared with regular adjuvanted conjugate vaccines and a Th1 immune response, suitable for cancer treatment. MPLA can be combined with other TLR agonists to induce dendritic cell maturation and obtain synergistic effects such as reduced PD-1 expression and better effector T-cell function [83,84,85]. α-GalCer activates iNKT cells, a subset of T-cells with natural killer properties recognizing MHC-like molecule CD1. While their role is poorly understood, iNKT cell activation correlates with good cancer prognosis [86]. α-GalCer was used as a carrier for sTn and for Tn, which outperformed leading Tn-CRM197 conjugates [22,87]. In a recent breakthrough, Tn was incorporated in a three-component vaccine containing both MPLA and α-GalCer analog KRN-7000 with yet higher antibody titers than either Tn-MPLA or Tn-KRN-7000 and excellent Tn specificity, clearly demonstrating the benefits of monomolecular adjuvanted constructs [88].

### 3.5. Multivalent Presentation and Clusters

The heterogeneity of TACA expression both between patients and between cells within a tumor naturally brings the idea of vaccines targeting multiple TACAs. This could yield vaccines suitable for broad patient populations without prescreening for tumor expression and reduce risks of tumor cells losing TACA expression as a protective measure. Livingston, a pioneer of anti-TACA vaccine developing TACA-KLH conjugates, hence mixed six KLH conjugates into one formulation, injected eight times into patients [89]. The hexavalent vaccine performed poorly, with antibody titers decreased or absent for all six TACAs in humans, a surprise, as this approach is successful in 20-valent vaccines in bacteria (Prevnar 20). This is probably explained by the slow build-up of an immune response towards the large amount of carrier injected with the vaccine over the course of an already long treatment regimen (eight injections) required for anticancer vaccines_,_ a phenomenon known as carrier-induced epitope suppression (CIES).

### 3.6. Carrier-Induced Epitope Suppression

CIES encompasses mechanisms by which the carrier of a conjugate vaccine interferes with the immune response towards the vaccine. It was discovered when Herzenberg et al. tried to treat mice pre-immunized with KLH with a dinitrophenyl (DNP)-KLH vaccine. Surprisingly, IgG towards DNP decreased, indicating that pre-existing antibodies towards KLH neutralized the DNP-KLH construct before immune presentation [90,91,92]. Following this mechanism, increasing the amount of protein carrier or using longer injection schedules builds up immunity towards the carrier, and hence reduces vaccine efficacy. A second CIES mechanism has since been discovered, active from the first encounter with a carrier. During immunization with a protein carrier, a large portion of the antibodies target the carrier rather than the antigen, and this fraction increases with the immunogenicity of the carrier [91]. The effect can be mitigated by combining several monovalent conjugates into one polyvalent construct to reduce the total amount of carrier protein in the vaccine. This approach also forms antigen clusters, closer to the natural cellular presentation of TACAs. To that end, Danishefsky’s group assembled five TACAs on a single peptide, conjugated to KLH. A phase I study demonstrated a solid immune response towards most antigens in most patients [93,94]. Considerable research efforts are also underway to develop a new generation of less immunodominant carriers to reduce or eliminate CIES (Section 4).

The same immune suppression is observed with the linkers used to attach the antigen to the carrier. A commonly used maleimide-containing linker was shown by Boons’ group to elicit a strong immune response. The maleimide of a Le^y^-KLH glycoconjugate vaccine was replaced by 3-(bromoacetamido)propionate, a smaller and more flexible linker. This led to increased IgG antibody response against Le^y^ [95].

## 4. Alternative Vaccine Carriers

**Figure 2:**
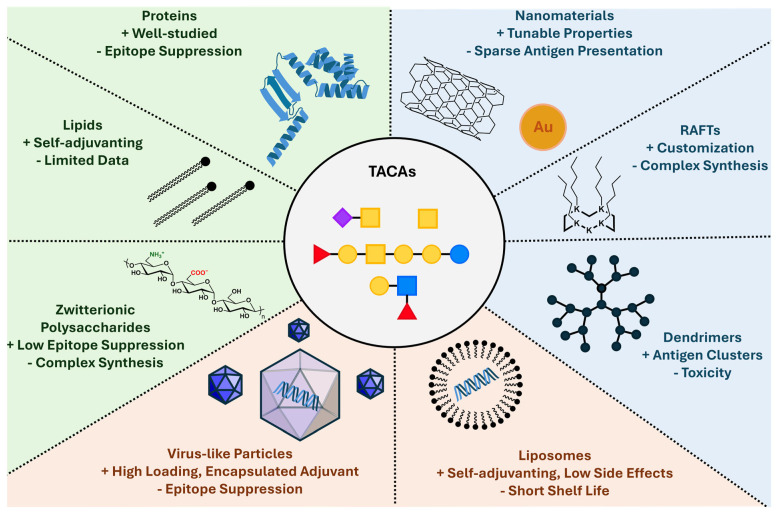
Overview of TACA vaccine carriers. The low immunogenicity of TACAs requires carriers with low epitope suppression, high loadings, targeted delivery or precise biologically accurate presentation. Several carrier platforms are under investigation to replace traditional protein carriers. Carbohydrate are drawn using the symbol nomenclature for glycans.

The most logical way to overcome the limits of protein carriers remains the exploration of new carrier technologies. These creative approaches offer high versatility and loadings, more natural antigen presentation or lower carrier-induced suppression (Figure 2).

### 4.1. Virus-like Particles

Virus-like particles (VLPs) are self-assembled structures composed of viral proteins; they range from 20 to 200 nm, typically assemble as icosahedra or rods, and mimic viruses to invoke an immune response. VLPs are particularly advantageous as carriers due to their size, allowing them to freely drain into lymph nodes, and their ability to promote the activation of CD4^+^, CD8^+^ and NKT cells [96]. VLPs can encapsulate nucleic acid oligomers, reportedly inducing T-cell independent class switching to IgG. This still poorly elucidated mechanism could come from the cross-linking of the BCR and TLR receptor by RNA strands [97,98,99,100].

VLPs have been harnessed by Huang’s group, using Qβ. This widely studied 28 nm icosahedral bacteriophage-derived VLP composed of 180 14.3 kDa protein subunits is well-known for its strong T-cell dependent response [101]. Qβ has been functionalized with glycopeptides MUC1-Tf and MUC1-sTn [102,103]. The construct, adjuvanted with MPLA, resulted in exceptionally high antibody titers.

### 4.2. Liposomes

Liposomes are a highly versatile drug delivery platform, allowing for the complete customization of size, physicochemical properties and antigen presentation mode. Their size allows them to drain to lymph nodes, eliciting an immune response through internalization by APCs, direct interaction with B-cell receptors, or cross presentation by MHCI [104]. As such, liposomes are currently used in FDA-approved vaccines Shingrix (shingles, subunit vaccine) [105], Comirnaty (COVID-19, mRNA) [106] and Spikevax (COVID-19, mRNA) [107], Mosquirix (malaria, subunit/conjugate vaccine) [108] among others. Liposomes have hence been tried as a delivery platform for conjugate vaccines. A Tn-MUC1 vaccine adjuvanted with CpG DNA, a TLR agonist, was hence loaded into liposomes assembled from Pam3CSK4-K5, a TLR-2 agonist, self-assembling into 10–130 nm liposomes. This heavily adjuvanted vaccine induced macrophage activity, isotype switching and, when paired with a CD4^+^ epitope, led to CD4^+^ T-cell activation [109]. Similarly, Tn was conjugated to cholesterol to form self-assembling liposomes ranging from 120 to 220 nm and loaded with CpG motifs. The smallest liposomes induced high levels of isotype switching and a T-dependent response [110]. Finally, a vaccine targeting ganglioside GM3 was designed using very small size proteoliposomes (VSSPs) derived from *Neisseria meningitidis* outer membrane vesicles (OMVs). The resulting vaccine proved excellent at breaking tolerance towards GM3 despite its moderate expression on healthy cells. The resulting GlycoVax GM3 vaccine can be processed by dendritic cells even in immunocompromised patients [111,112], yields high antibody titers and improves survival, as demonstrated in a series of phase II and III clinical trials in Cuba targeting breast, lung and skin cancers (RPCEC00000220, RPCEC00000070, RPCEC00000223 and RPCEC00000224) [113,114]. OMVs have also been functionalized with the Tn and sTn antigen, yielding excellent IgG1, IgG2a and IgG2b titers in mice [115]. The method required a finely controllable thiol-based functionalization of OMVs [116].

### 4.3. Other Nanoparticles

Several other biomolecule-based nanoparticle carriers are used in TACA vaccines. Synthetic homogeneous nanosheets, nanotubes and micelles were assembled with two amphiphilic block copolymers comprising sarcosine (*N*-methylglycine), L-lactic acid, D-Leucine and aminoisobutyric acid in different ratios. The nanomaterials, functionalized with Le^y^, did not induce Lewis-specific IgGs, probably due to a low antigen density [117]. Dextran nanoparticles, used as potential protein mimics with small controllable size (ca. 10 nm), loaded with Tn antigen induced a strong innate immune response, with no toxicity. The lack of adaptive immune response to the construct could be easily mitigated in the future by incorporating a T-cell epitope in the vaccine [118].

Dykman’s group discovered the immunogenicity of colloidal gold as a carrier when used in combination with various antigens [119,120]. Gold nanoparticles (AuNPs) have since demonstrated strong immunostimulatory properties, leading to their use as both a carrier and an adjuvant [121]. AuNPs are inert and have tunable electromagnetic absorption properties, enabling synergies with radiotherapy [122]. A TF-MUC4 antigen coated on AuNPs elicited IgGs in a mouse model, although a complement-activating adjuvant was still required [123]. Similarly, a Tn-MUC1 antigen conjugated to PEGylated AuNPs invoked strong Th1- and Th2-mediated responses that produced IgG specific for Tn-expressing MCF-7 breast cancer cells [124]. A recent strategy used β-1,3-glucans as both an APC-promoting adjuvant and a reducing agent to coat AuNPs. The MUC4-Tf antigen was conjugated to these glucan-coated AuNPs, leading to a strong antibody response consisting of mixed Th1-, Th2-, and Th17-mediated responses and a favorable cytokine profile, including a low expression of Il-10 [125].

In addition to AuNPs, other inorganic nanoparticles have been explored. Iron oxide and silica nanoparticles have been used to mimic the natural multivalent presentation of TACAs, as illustrated by their conjugation to a TnThr mimic, leading to strong APC activation [126].

### 4.4. Dendrimers

Dendrimers are hyperbranched, highly tunable and controllable polymers. These well-defined spherical particles have received growing attention as vaccine carriers thanks to their ease of derivatization into multivalent glycan-based vaccines [127,128]. Originally reported in 1985, dendrimers can now be synthesized from a large variety of building blocks, including poly(amidoamine) (PAMAM), poly(L-lysine) (PLL) and poly(propylene imine) (PPI), and are easily cross-linked or converted to nanogels, tuning finely loading, delivery and toxicity [129,130].

Dendrimers have been especially useful to force cluster presentation of TACAs by vaccines, closer to cell surface presentation than typical glycoconjugates. To this end, Tn-MUC1 was conjugated to a β-cyclodextrin dendrimer core. The resulting heptavalent vaccine yielded antibodies with five times higher avidity than those obtained from a monovalent version of the vaccine [131].

Finally, multiple antigen glycopeptide (MAG) is a dendrimer that has received special attention in the field of TACA-based anti-tumor vaccines. The first MAG glycoconjugate was a tetramer of a poliovirus-derived CD4^+^ T-cell peptide epitope with Tn conjugated to each peptide subunit [132]. This strategy was used to conjugate Tn in clusters of three to mimic mucin glycosylation patterns. The resulting MAG-Tn3 vaccine performed significantly better than an analogous Tn-KLH conjugate in the production and maintenance of anti-Tn antibodies, immune response specificity, and establishment of immunological memory. Similar results were obtained in a macaque model, including a high anti-Tn IgG titer and NKT-cell killing of tumor cells [133]. Eventually, the NKT-cell activity induced by MAG-Tn3 was confirmed to be a result of antibody-dependent cell cytotoxicity [134]. MAG-Tn3 underwent phase I clinical trials, where it induced a strong anti-Tn IgG response, high IFNγ and Il-2 secretion, and complement-dependent cytotoxicity [135].

Some carriers can be combined to utilize the strengths of diverse strategies. Dendrimers have recently demonstrated the ability to entrap, stabilize and assemble AuNPs. AuNPs mask the toxic amine groups of PAMAM dendrimers and, due to their aforementioned versatility, can act as an adjuvant or aid in combinatorial therapy [136].

### 4.5. Regioselectively Addressable Functionalized Templates (RAFTs)

Regioselectively addressable functionalized templates (RAFTs), pioneered by Dumy’s group, are dendrimers with cyclic lysine-rich decapeptide cores. The protected lysine residues of the RAFT decapeptide can be orthogonally deprotected and functionalized with unique side chains to easily prepare well-defined multivalent constructs [137].

The technology was applied to Tn by the same group ten years later. Of the five lysine residues, four were functionalized with Tn, and the fifth with a poliovirus-derived CD4^+^ T-cell epitope [138]. Using the same conjugation strategy, researchers in France conjugated four sTn and PADRE, a synthetic CD4^+^ T-cell peptide epitope, to RAFT subunits [139]. These researchers used these subunits, as well as RAFT subunits containing Tn, TF, GM2, and Globo H antigens, to synthesize RAFTs with high heterovalency to mimic the microheterogeneity found in the glycocalyx of metastatic cells [140]. The RAFTS were adjuvanted with QS-21. The resulting vaccines revealed excellent isotype switching and a balanced Th1/Th2 immune response with high IgG2c titers, an antibody subtype that strongly influences CDC and ADCC [141].

### 4.6. Zwitterionic Polysaccharides

**Figure 3:**
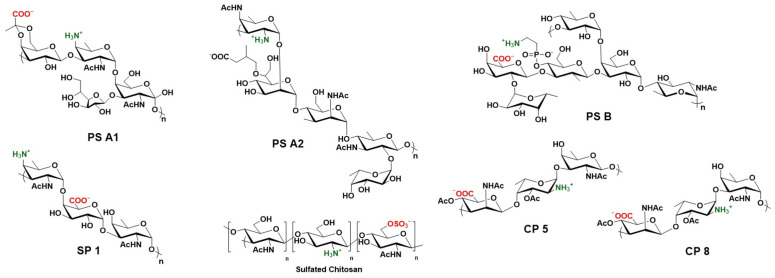
Known natural and synthetic zwitterionic polysaccharides.

Zwitterionic polysaccharides (ZPSs) are rare bacterial capsular polysaccharides that display alternating charges on adjacent monosaccharides (Figure 3). Unlike other carbohydrates, they can be processed by MHCII and trigger CD4^+^ T-cells, immune memory and antibody isotype switching to high-affinity IgG1, IgG2a, IgG3 and IgE without a carrier protein [142,143]. This has been linked to their alternating charges, forcing a helical conformation not unlike protein α-helices, allowing binding to MHCII [144].

These unique properties make them an interesting alternative to protein carriers, following the same immune presentation pathways but displaying much lower immunity towards the carrier, hence reducing carrier-induced epitope suppression [145].

Among them, PS A1 from the capsule of *Bacteroides fragilis* (ATCC 25285/NCTC 9343) has been extensively studied by our group due to both an attractive acyclic diol handle for conjugation and its TLR-2 agonist properties, providing adjuvant-like behavior [146]. TACAs Tn, sTn, sTf, GM3, and Globo H conjugated to PS A1 have all induced high IgG titers and a strong ability to elicit complement against cancer cell lines [91,147,148], and monoclonal antibodies raised against Tn reduced the progression of triple-negative breast cancer by 30% in immunodeficient mice grafted in the thigh with a human breast cancer cell line [149]. Recent work shows that PS A1 vaccines containing a poliovirus-derived MHCI epitope can engage potent CD8^+^ T-cell responses [150].

Harnessing the properties of zwitterionic polysaccharides, several methods have been developed to turn T-cell independent polysaccharides like chitosan into T-cell dependent antigens by introducing balanced positive and negative charges. The resulting constructs have been tested as vaccine adjuvants with good results [151,152].

## 5. Conclusions and Perspectives

To overcome past clinical trial failures faced by anti-TACA glycoconjugate vaccines, a paradigm shift is needed towards new vaccine technologies improving immunogenicity towards the antigen or forcing more biologically relevant antigen conformations.

This work is currently divided into two major fields of research: improving the targeted antigens to provide potent and specific immune responses and improving the carriers to reduce carrier interference spanning from the long treatment regimens required for cancer treatments.

On the antigen side, the development of carbohydrate synthesis has permitted the development of well-defined synthetic antigens, paving the way for new findings in the structure–activity relationship of TACAs, the importance of antigen conformation, and the refinement of conjugation methods to vaccine carriers.

On the carrier side, a new generation of versatile carriers has been investigated to solve the problems associated with protein-carried glycoconjugate vaccines, particularly carrier-induced epitope suppression, while retaining T-cell dependence. These alternative carriers demonstrate high multi-functionality due to their synthetic nature, lower toxicity, and opportunity for precise antigen delivery. While more versatile carriers with a modulated immunogenicity are advantageous, it remains important to develop T-cell dependent glycoconjugates to ensure an effective immune response towards T-cell independent TACAs, a requirement in anticancer vaccines.

The field is now racing towards a new series of clinical trial challenges. While pre-clinical testing and clinical trials for alternative carriers have yielded promising results demonstrating the maturity of these technologies, it is time to learn from three decades of research and combine technologies now proven effective towards even more effective vaccines by (i) exploring multivalency, either through the addition of multiple copies of antigens or the presentation of different TACAs, to mimic cancer cell presentation; (ii) learning from efforts on antigen structure–activity studies, using novel higher immunogenicity mimics of natural antigens; and (iii) using low-immunogenicity high-loading carriers capable of eliciting strong and sustained immune responses towards the target antigen with low carrier interference.

While current approaches will result in highly improved immunogenicity, it remains to be seen whether this will be sufficient to bypass the immunosuppressive tumor microenvironment, and whether these targeted therapies can be evaded by cancer cells losing expression of the targeted antigen(s). To overcome the latter, a custom treatment for each patient would be beneficial. In-depth screening of TACAs expressed by a specific patient, combined with robust, reproducible, modular carriers like RAFTs or ZPSs could provide personalized multivalent vaccines yielding highly targeted, long-lasting immune responses in patients while keeping carrier amounts, and hence carrier interference, to a minimum. Incorporating antigen mimics, more immunogenic than their natural counterparts, would further enhance immune response with limited drawbacks.

Combination of the methods described here, together with immune stimulants like granulocyte-macrophage colony-stimulating factors or interleukins to weaken immune evasion and with existing immunotherapy and radiotherapy techniques, is likely required. Ongoing and future clinical trials will indicate whether anti-TACAs vaccines have finally reached the immune thresholds required for efficacy or whether further developments are still required.

## Data Availability

No new data were created or analyzed in this study. Data sharing is not applicable to this article.

## Abbreviations

The following abbreviations are used in this manuscript:

ADCC	Antibody-dependent cell cytotoxicity
APC	Antigen-presenting cell
ATCC	American type culture collection
AuNP	Gold nanoparticle
BCR	B-cell receptor
BSA	Bovine serum albumin
CAR	Chimeric antigen receptor
CDC	Complement-dependent cytotoxicity
CIES	Carrier-induced epitope suppression
DC	Dendritic cell
DC-SIGN	Dendritic cell-specific intercellular adhesion molecule-3-grabbing non-integrin
DNP	Dinitrophenyl
HLA	Human leukocyte antigen
iNKT	Invariant natural killer T-cell
KLH	Keyhole limpet hemocyanin
MAG	Multiple antigen glycopeptide
MHC	Major histocompatibility complex
MPLA	Monophosphoryl lipid A
MUC1	Mucin 1
NCI	National Cancer Institute
NKT	Natural killer T-cell
OMV	Outer membrane vesicle
PAMAM	Poly(amidoamine)
PD	Programmed death
PEG	Polyethylene glycol
PLL	Poly-(L-lysine)
PPI	Poly(propylene imine)
PS A1	Polysaccharide A1
RAFT	Regioselectively addressable functionalized template
SSEA	Stage-specific embryonic antigen
TACA	Tumor-associated carbohydrate antigen
TLR	Toll-like receptor
VLP	Virus-like particle
VSSP	Very small size proteoliposomes
ZPS	Zwitterionic polysaccharide
